# Priming With Toll-Like Receptor 3 Agonist Poly(I:C) Enhances Content of Innate Immune Defense Proteins but Not MicroRNAs in Human Mesenchymal Stem Cell-Derived Extracellular Vesicles

**DOI:** 10.3389/fcell.2021.676356

**Published:** 2021-05-24

**Authors:** Lisa M. Pierce, Wendy E. Kurata

**Affiliations:** Department of Clinical Investigation, Tripler Army Medical Center, Honolulu, HI, United States

**Keywords:** mesenchymal stem cells, extracellular vesicles, poly(I:C), priming, innate immunity, miRNA, host defense proteins, TLR3 agonist

## Abstract

Mesenchymal stem cells (MSCs) help fight infection by promoting direct bacterial killing or indirectly by modulating the acute phase response, thereby decreasing tissue injury. Recent evidence suggests that extracellular vesicles (EVs) released from MSCs retain antimicrobial characteristics that may be enhanced by pretreatment of parent MSCs with the toll-like receptor 3 (TLR3) agonist poly(I:C). Our aim was to determine whether poly(I:C) priming can modify EV content of miRNAs and/or proteins to gain insight into the molecular mechanisms of their enhanced antimicrobial function. Human bone marrow-derived MSCs were cultured with or without 1 μg/ml poly(I:C) for 1 h and then conditioned media was collected after 64 h of culture in EV-depleted media. Mass spectrometry and small RNA next-generation sequencing were performed to compare proteomic and miRNA profiles. Poly(I:C) priming resulted in 49 upregulated EV proteins, with 21 known to be important in host defense and innate immunity. In contrast, EV miRNA content was not significantly altered. Functional annotation clustering analysis revealed enrichment in biological processes and pathways including negative regulation of endopeptidase activity, acute phase, complement and coagulation cascades, innate immunity, immune response, and *Staphylococcus aureus* infection. Several antimicrobial peptides identified in EVs remained unaltered by poly(I:C) priming, including dermcidin, lactoferrin, lipocalin 1, lysozyme C, neutrophil defensin 1, S100A7 (psoriasin), S100A8/A9 (calprotectin), and histone H4. Although TLR3 activation of MSCs improves the proteomic profile of EVs, further investigation is needed to determine the relative importance of particular functional EV proteins and their activated signaling pathways following EV interaction with immune cells.

## Introduction

Mesenchymal stromal (stem) cells (MSCs) are multipotent cells isolated from bone marrow, adipose tissue, umbilical cord, and other tissues that are widely used in translational applications (De Castro et al., 2019). MSCs are considered immune privileged because they express low levels of human leukocyte antigen (HLA) class I molecules and often lack expression of HLA class II molecules, making them useful for cell therapy purposes (Marrazzo et al., 2019). MSCs have been shown to benefit wound healing not only by promoting tissue regeneration and increasing the recruitment of macrophages and endothelial cells into the wound but also by possessing immunomodulatory and antimicrobial activity against both Gram-negative and Gram-positive pathogens (Alcayaga-Miranda et al., 2017; Marrazzo et al., 2019). In preclinical models of sepsis, acute respiratory distress syndrome, and surgical mesh infection, several investigators found that MSCs enhanced bacterial clearance, decreased inflammation, and improved survival (Mei et al., 2010; Gupta et al., 2012; Yuan et al., 2014; Criman et al., 2016; Zheng et al., 2018).

MSCs help fight infection by promoting direct bacterial killing or indirectly by modulating the acute phase response, thereby decreasing tissue injury (Marrazzo et al., 2019). MSCs have been shown to secrete antimicrobial peptides (AMPs) such as cathelicidin (hCAP-18/LL-37), beta-defensin 2, lipocalin 2, and keratinocyte growth factor (Krasnodembskaya et al., 2010; Lee et al., 2013; Sung et al., 2016). In addition, conditioned media from human MSCs was discovered to inhibit *Pseudomonas aeruginosa* biofilm formation, demonstrating that MSCs can also secrete factors that can prevent or reduce the growth of biofilms (Wood et al., 2018). Biofilms are produced when a cluster of organisms attach to a surface and secrete an extracellular polysaccharide matrix that serves as a protective barrier against conventional antibiotics and host defenses (Khatoon et al., 2018).

The beneficial effects of stem cell-based therapies have been proposed to be mediated predominantly by paracrine activity, to include soluble factors and extracellular vesicles (EVs) secreted from MSCs (Phinney and Pittenger, 2017; Zheng et al., 2018; Seo et al., 2019). EVs are composed of different types of vesicles including exosomes (40–200 nm), which are of endosomal origin, and microvesicles (150–1,000 nm), which directly bud from the cell membrane. Because current isolation methods cannot physically separate exosomes from small microvesicles, the term “EVs” refers to vesicles in the size range of exosomes. MSCs have been shown to release EVs that can transfer microRNAs (miRNAs), mRNAs, proteins, and lipids to injured tissues that are capable of modulating gene expression and function of recipient cells (Phinney and Pittenger, 2017; Zheng et al., 2018; Seo et al., 2019). Considered to be theoretically safer than MSCs as a cell-free therapeutic approach, EVs have been shown to be as efficacious in several disease models as their parent MSCs and exhibit neuro-protective, cardio-protective, renal-protective, and immunosuppressive activities (Yin et al., 2019). Immunomodulatory and immunostimulatory properties of MSC-derived EVs likely depend on selectively packaged functional miRNAs and AMPs shuttled by the EVs (Alcayaga-Miranda et al., 2017; Seo et al., 2019). MiRNAs are recognized as being an integral part of the host immune response to efficiently fight infection by various pathogens, including bacteria, viruses, and parasites (Aguilar et al., 2019).

Preconditioning or “priming” has been proposed as an important strategy to prime the antimicrobial and immunological properties of MSCs *ex vivo* to enhance their potential for treating bacterial infection (Waterman et al., 2010). Studies have shown that stimulation of specific toll-like receptors (TLRs) affects the immune modulating responses of MSCs. For example, TLR3 stimulation of human MSCs establishes their immunosuppressive effects, while TLR4 activation elicits proinflammatory effects (Waterman et al., 2010). MSCs primed with the TLR3 ligand polyinosinic polycytidylic acid [poly(I:C)] were able to suppress and eradicate chronic *Staphylococcus aureus* biofilm infection in mice and improved healing in dogs with spontaneous chronic multidrug-resistant wound infections (Johnson et al., 2017). TLR3-activated human MSCs were also shown to more effectively reduce sepsis-induced inflammation and organ dysfunction and improve overall survival in a mouse model of polymicrobial sepsis (Zhao et al., 2014).

The beneficial effects of poly(I:C) priming of parent MSCs appear to be replicated in their secreted EVs. Recent studies investigating severe bacterial pneumonia in both a preclinical mouse model and in an *ex vivo* perfused human lung demonstrated that preconditioning human bone marrow-derived MSCs with poly(I:C) also enhanced the antibacterial activity of the MSC-EVs, suggesting that MSC activation improves the secretion profile of EVs (Monsel et al., 2015; Park et al., 2019). Monocytes exposed to poly(I:C)-primed MSC-EVs exhibited enhanced phagocytosis of *Escherichia coli*, increased expression of cyclooxygenase-2 mRNA (important for the synthesis of prostaglandin E2, the key molecule in the immunomodulatory function of MSCs), decreased TNF-α secretion, and increased IL-10 secretion characteristic of an M2 immunomodulatory phenotype (Monsel et al., 2015).

Although altered immunomodulatory miRNA and protein expression profiles have been detected in EVs released from MSCs after priming with other agents such as interferon gamma (IFNγ), no published studies have determined whether MSC activation with poly(I:C) can increase the EV content of miRNAs and/or proteins important in host defense and innate immunity (Zhao et al., 2017; Marinaro et al., 2019; Peltzer et al., 2020). Therefore, the purpose of this study was to investigate the miRNA and proteomic signatures of poly(I:C)-primed MSC-EVs to gain insight into the molecular mechanisms of their enhanced antimicrobial function after TLR3 activation.

## Materials and Methods

### Cell Culture and Priming

Human bone marrow-derived MSCs purchased from Lonza were expanded under standard culture conditions in a complete stem cell medium (Poietics MSCGM Mesenchymal Stem Cell Growth Medium BulletKit, Lonza). These cells have been verified by Lonza to be multipotent, capable of differentiating into adipocytes, osteoblasts, and chondrocytes and analyzed using different CD markers to confirm that they meet MSC criteria as defined by the International Society for Cellular Therapy (Dominici et al., 2006). For MSC priming, cells (at passages 4–5 and 60–80% confluent) were incubated for 1 h in Dulbecco’s Modified Eagle’s Medium (DMEM) High Glucose/1 × GlutaMAX (Thermo Fisher Scientific) + 0.5% bovine serum albumin + 1 μg/ml poly(I:C) (MilliporeSigma) as described (Waterman et al., 2010; Monsel et al., 2015). Control MSCs underwent the same treatment but without poly(I:C). Cells were then rinsed with phosphate buffered saline (PBS) and media was replaced with DMEM High Glucose without Phenol Red/1 × GlutaMAX containing 5% exosome-depleted fetal bovine serum (Exo-FBS, System Biosciences [SBI]). Cells were cultured an additional 64 h and then the media was collected and stored at –80°C until EV isolation. We verified that these conditions did not reduce cell viability as detected by a Live/Dead viability/cytotoxicity kit (Thermo Fisher Scientific) following the manufacturer’s protocol (not shown). Experiments were performed in three replicates using MSCs from one donor.

### EV Characterization

Characterization of unprimed MSC-EVs (morphology, particle size analysis, and exosome marker detection) was performed by Creative Bioarray utilizing their Exosome Isolation and Identification Service. MSC-EVs were isolated by the ultracentrifugation method using a Hitachi CP100MX ultracentrifuge, resuspended in PBS, and stored at -80°C until use.

*Transmission electron microscopy (TEM)*. For morphological analysis, TEM was performed on EVs with a Hitachi HT-7700 microscope at 80 kV. Samples were placed onto copper grids for 1 min at room temperature, excess solution was removed with filter paper, uranyl acetate solution was added, and the copper grids were dried for several minutes before imaging.

*EV particle size analysis*. To determine size distribution and concentration, 10 μl of the sample was detected with a Flow NanoAnalyzer (NanoFCM, N30E).

*Exosome marker detection by nano-flow cytometry*. For phenotyping of EVs, 20 μl of fluorescent labeling antibodies against surface proteins CD9 (BD Biosciences), CD81 (BD Biosciences), and IgG as a control (Biolegend) was added into 30 μl of the EV sample with a particle concentration of 2.2 × 10^8^ particles/ml. The mixture was incubated at 37°C for 30 min and then washed twice with 1 ml of PBS followed by ultracentrifugation at 110,000 × *g* for 70 min at 4°C. The pellet was resuspended in 50 μl of PBS for nano-flow cytometry analysis.

### Proteomic Analysis

Proteomic analysis of unprimed (control) MSC-EVs and poly(I:C) MSC-EVs was performed by SBI utilizing SBI’s ExoMS Total Exosome Protein Profiling Service. Briefly, EVs were isolated using an affinity purification strategy to remove free protein. EV proteins were processed for gel-based extraction and trypsinization to generate peptidic libraries for liquid chromatography–tandem mass spectrometry (LC-MS/MS). Samples were analyzed by nano-LC-MS/MS (Dionex UltiMate 3000 RLSCnano System) interfaced with an Orbitrap Eclipse Tribrid mass spectrometer (Thermo Fisher Scientific). Peptides were loaded onto a fused silica trap column (Acclaim PepMap 100, Thermo Fisher Scientific) and eluted over a 75-μm analytical column (Waters Co., nanoEase M/Z peptide BEH C18 column, Thermo Fisher Scientific) at 300 nl/min. The scan sequence began with an MS1 spectrum (Orbitrap analysis, resolution 120,000, scan range from m/z 350–1,600, automatic gain control target 1E6, and maximum injection time 100 ms). Parent masses were isolated in the quadrupole with an isolation window of 1.4 m/z, automatic gain control target 1E5, and fragmented with higher-energy collisional dissociation with a normalized collision energy of 30%. The fragments were scanned in Orbitrap with a resolution of 30,000.

Peptide signatures were analyzed with Proteome Discoverer 2.4 (Thermo Fisher Scientific) and mapped to a database of known protein sequences (SwissProt Human) and to a database consisting of common lab contaminants. The Percolator node in the processing workflow was used for results validation. A concatenated reverse database was used as a target-decoy search strategy. Data were filtered using 1% protein and peptide false discovery rate and required at least two unique peptides per protein. A label-free quantification method based on the Minora algorithm was used for chromatographic feature detection, and protein abundance was calculated using the summed abundance value of all unique + razor peptides. Normalization was performed using the summed abundance of all peptides for that sample.

To evaluate proteomic alterations between poly(I:C) MSC-EVs and control MSC-EVs, proteins present in all samples per group and showing greater than a fivefold change were considered differentially expressed. Identified proteins were analyzed for enrichment in terms of their gene ontology (GO) categories and Kyoto Encyclopedia of Genes and Genomes (KEGG) pathways using the Functional Annotation Clustering Tool in the Database for Annotation, Visualization and Integrated Discovery (DAVID Bioinformatics Resource 6.8) using default settings (Huang et al., 2009a,b). Enrichment analysis in DAVID used a modified Fisher Exact test to determine whether proteins were enriched in the annotation categories, and enrichment statistics were adjusted for multiple hypothesis testing by the Benjamini correction.

### RNA Sequencing Analysis

Next-generation sequencing of control and poly(I:C) MSC-EVs was performed by SBI utilizing SBI’s Exosome RNA NGS Service as reported previously (Mayo et al., 2019). Briefly, EVs were isolated using SBI’s ExoQuick precipitation method, and RNA was extracted using the SeraMir Exosome RNA Purification Column Kit (SBI). The purified, size-selected library (140-bp to 300-bp region) was quantified with the High Sensitivity DNA 1000 Screen Tape Kit (Agilent Technologies) and the TailorMix HT1 qPCR Assay (SeqMatic), followed by a NextSeq High Output single-end sequencing run at SR75 using NextSeq 500/550 High Output v2 Kit (Illumina) at an average depth of 13 million reads per sample. Data analysis was conducted using the Banana Slug Exosome RNA-seq Analysis platform using the human reference GRCh37/hg19 assembly (UCSC Genome Bioinformatics). The DESeq package was used to identify candidate differentially expressed miRNAs with significance considered at > twofold change and *p* < 0.05 (adjusted for multiple comparisons).

To determine the biological significance of EV miRNAs, TargetScan Release 7.2 was used to generate a list of predicted miRNA targets (Agarwal et al., 2015). A cutoff of ≤ –0.3 cumulative weighted context + + score was used to exclude weak predictions. Predicted miRNA target genes were then analyzed for enrichment in terms of their KEGG pathways within DAVID, and enrichment statistics were adjusted for multiple hypothesis testing by the Benjamini correction.

## Results

### EV Characterization

**FIGURE 1:**
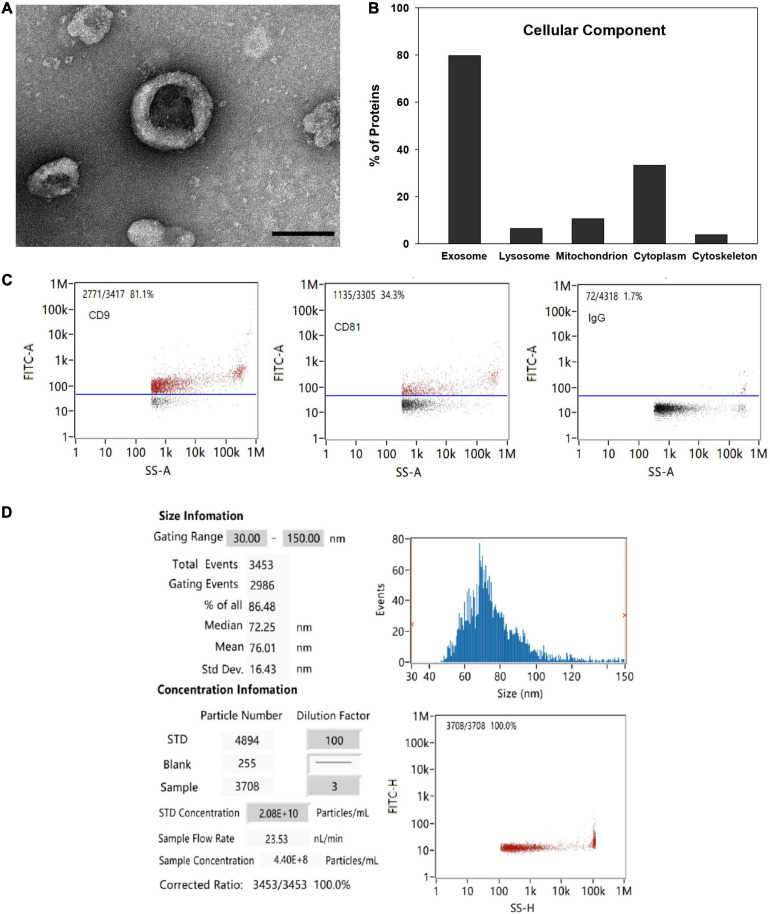
EV characterization. **(A)** Representative TEM image of MSC-EV preparation (bar = 100 nm). **(B)** Functional gene ontology enrichment analysis of EV proteins identified by mass spectrometry demonstrating distribution of proteins among cellular components. **(C)** Expression of exosomal markers CD9 and CD81 detected by nano-flow cytometry. **(D)** EV particle size and concentration analysis detected with a Flow NanoAnalyzer.

TEM analysis showed the typical cup-shaped morphology of the MSC-EVs with a central depression characteristic of EVs isolated by ultracentrifugation under TEM (Figure 1A). Functional GO enrichment analysis of proteins identified by mass spectrometry using DAVID software revealed that approximately 79.7% of identified proteins were associated with the GO term extracellular exosome (Benjamini *p* value 2.78 × 10^–156^; Figure 1B). The percentage of EVs expressing exosomal markers CD9 and CD81 as detected by nano-flow cytometry was 81.1 and 34.3%, respectively (Figure 1C). The average particle size was 76 nm, and 86.5% of all detected particles were in the range of 30–150 nm (exosome size range; Figure 1D).

### Protein Content of MSC-EVs and Poly(I:C) MSC-EVs

In total, 436 proteins were detected in the EV samples with high confidence. Among these 436 proteins, 238 proteins were present in all replicates (*n* = 3) of both groups (Supplementary Table 1). Only one protein, complement C1r subcomponent, was unique to poly(I:C) MSC-EVs, while three proteins were unique to control MSC-EVs (nebulin, Rab GDP dissociation inhibitor, and leukocyte elastase inhibitor). The most highly enriched biological processes and pathways common to EVs with or without poly(I:C) priming are listed in Supplementary Table 2. These include terms such as negative regulation of endopeptidase activity, proteasome core complex, NIK/NF-KB signaling, antigen processing and presentation of exogenous peptide antigen *via* MHC class I (TAP-dependent), complement and coagulation cascades, cell–cell adhesion, keratinocyte differentiation, innate immunity, antimicrobial, host–virus interaction, and cellular oxidant detoxification, which remained significant after the adjustment for multiple hypothesis testing using the conservative Benjamini correction.

**FIGURE 2:**
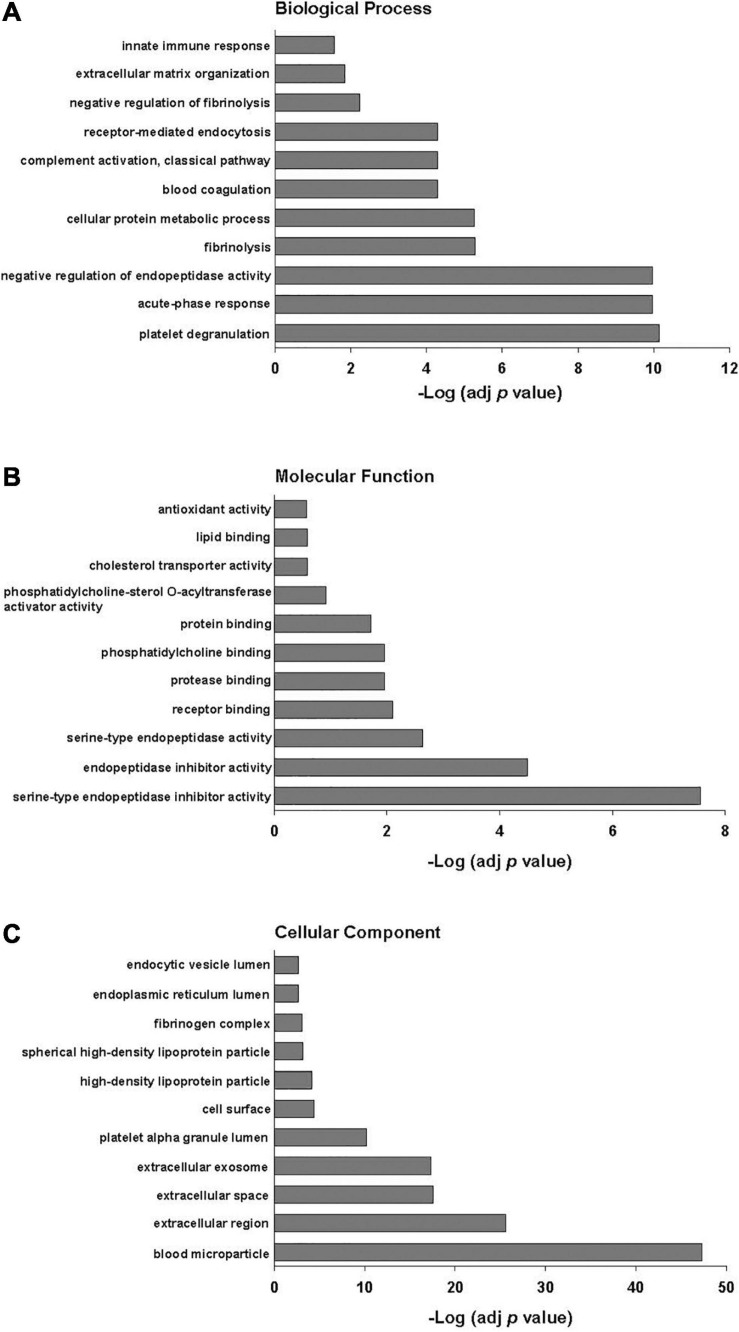
Gene ontology functional classification of proteins upregulated > fivefold in EVs after poly(I:C) priming of parent MSCs. **(A)** Biological process, **(B)** molecular function, and **(C)** cellular component. Enrichment *p* values were adjusted by Benjamini–Hochberg False Discovery Rate correction.

**FIGURE 3:**
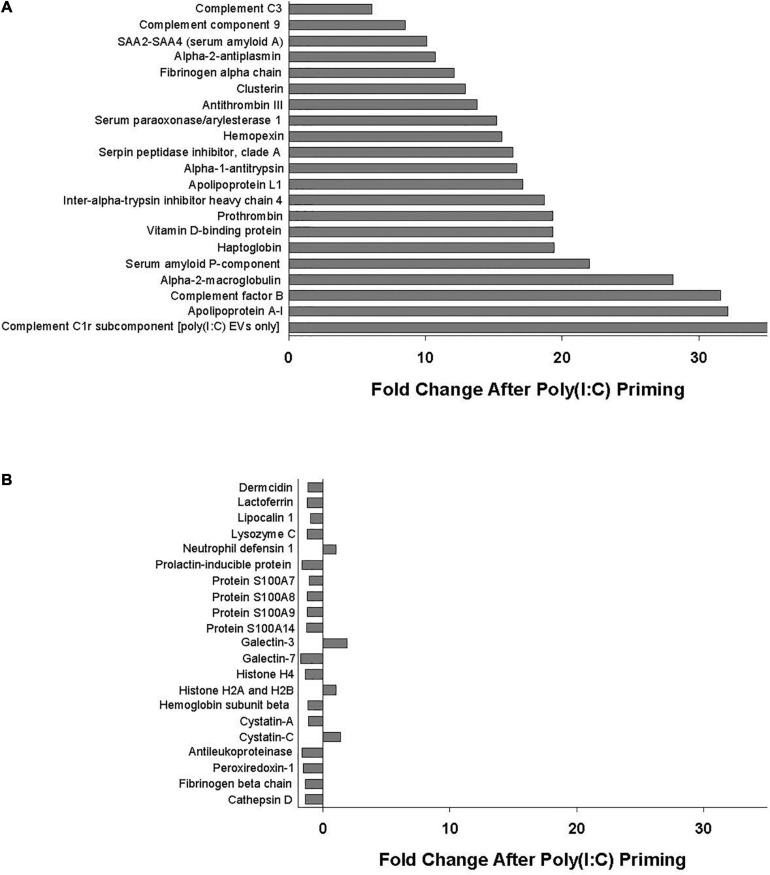
**(A)** Proteins important in host defense and innate immunity identified in all EV replicates whose levels were increased (>fivefold change) with poly(I:C) priming. **(B)** Antimicrobial peptides and defense proteins identified in all EV replicates that were unaltered (<twofold change) by poly(I:C) priming.

Poly(I:C) priming of parent MSCs resulted in 49 EV proteins that were upregulated > fivefold and only 6 proteins that were downregulated > fivefold, including those proteins unique to each group as discussed above. Functional annotation clustering analysis of upregulated EV proteins revealed enrichment in biological processes including acute-phase response, negative regulation of endopeptidase activity, complement activation, and innate immune response (Figure 2). Of the 49 proteins that were upregulated in poly(I:C) MSC-EVs, 21 are known to be important in host defense and innate immunity (Figure 3A).

Notably, several antimicrobial peptides that were identified in all EV samples remained unaltered (< twofold change) by poly(I:C) priming, including dermcidin, lactoferrin, lipocalin 1, lysozyme C, neutrophil defensin 1, S100A7 (psoriasin), S100A8/A9 (calprotectin), and histone H4, among others (Figure 3B).

### MiRNA Content of MSC-EVs and Poly(I:C) MSC-EVs

Exosome small RNA-seq analysis demonstrated that the EVs contain a broad range of RNA species, including non-coding RNAs (miRNAs, tRNAs, piwi-interacting RNAs, long non-coding RNAs, and other non-coding RNAs), coding sequences, and antisense transcripts (Supplementary Figure 1). An average of 567 and 473 known miRNAs were identified in control MSC-EVs and polyI:C MSC-EVs, respectively, with 250 miRNAs present in all replicates (*n* = 3) of both groups (Supplementary Table 3). No statistically significant differences in miRNA expression were identified between the groups.

**FIGURE 4:**
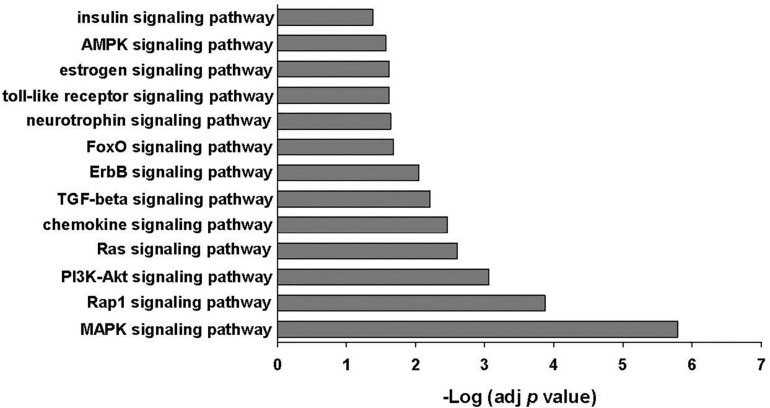
KEGG signaling pathways enriched among the genes predicted to be targeted by the 25 most abundant miRNAs identified in EVs ± poly(I:C). Enrichment *p* values were adjusted by Benjamini–Hochberg False Discovery Rate correction.

The 25 most abundant miRNAs identified in EVs released from MSCs with or without poly(I:C) priming made up 88–93% of all miRNAs present in the EVs (Supplementary Table 4). Fifteen of these miRNAs were also found in the top 23 most abundant miRNAs identified in human bone marrow-derived MSC-EVs by other investigators including miR-21-5p, miR-221-3p, let 7b-5p, let 7a-5p, miR-143-3p, miR-199a-3p, miR-199b-3p, let-7i-5p, miR-222-3p, let 7f-5p, miR-22-3p, miR-423-5p, miR-27b-3p, miR-100-5p, and miR-92a-3p (Baglio et al., 2015; Ferguson et al., 2018). Evaluation of putative target mRNAs using TargetScan Release 7.2 identified 2025 predicted gene targets of the top 25 most abundant miRNAs. Enrichment analysis of constructed gene sets in DAVID performed in order to gain functional insight identified several signaling pathways important in innate immunity including the MAPK signaling pathway, PI3K-Akt signaling pathway, chemokine signaling pathway, transforming growth factor beta signaling pathway, and the TLR signaling pathway, among others (Figure 4 and Supplementary Table 5).

## Discussion

Although antimicrobial effects of MSC-EVs have been documented, the underlying mechanisms of MSC-EVs are not well understood (Zheng et al., 2018). It is thought that immunomodulatory as well as immunostimulatory properties of MSC-EVs depend on selectively packaged miRNAs and proteins shuttled by the EVs in order to protect the body from invading pathogens (Alcayaga-Miranda et al., 2017; Mardpour et al., 2019; Seo et al., 2019). Findings from this study revealed that EVs released from bone marrow-derived MSCs contain miRNAs and proteins important in innate immunity and host defense that may be responsible for the antimicrobial and immunosuppressive effects of MSCs and MSC-EVs reported previously. Several AMPs known to have activities against various Gram-positive and Gram-negative bacteria, viruses, and fungi were identified in the EVs released from both unprimed and poly(I:C)-primed MSCs, supporting the hypothesis that EVs conserve the antimicrobial effects of MSCs in part through their AMP content (De Smet and Contreras, 2005; Alcayaga-Miranda et al., 2017; Patel and Akhtar, 2017; Mayo et al., 2019). Furthermore, we showed that preconditioning parent MSCs with poly(I:C) may enhance antimicrobial effects by upregulating several EV proteins important in complement and coagulation cascades, modulation of the acute phase response, and innate immunity.

Of the 49 proteins that were upregulated in poly(I:C) MSC-EVs, 21 (listed in Figure 3A) are known to be important in host defense and innate immunity including several members of the complement system such as C3, C9, complement factor B, and complement C1r subcomponent. The complement system is essential for antimicrobial defense, homeostasis, and immunomodulation and includes several proteins comprising components of the classical, alternative, and lectin pathways (Heesterbeek et al., 2018). Complement activation results in direct bacterial killing *via* large pore-forming complexes and rapid clearance of pathogens by immune cells (Heesterbeek et al., 2018). Synthesis of complement factor B, one of the most highly upregulated proteins in poly(I:C) MSC-EVs, was also shown to increase after poly(I:C) stimulation in colonic epithelial cells (Ostvik et al., 2014). Other proteins upregulated in poly(I:C) MSC-EVs include several members of the serpin superfamily of serine protease inhibitors known to regulate protease activity thereby reducing tissue damage and immune cell death (Mangan et al., 2008). Apolipoprotein A-I, a major component of the high-density lipoproteins and one of the most highly upregulated EV proteins, has been shown to exert antimicrobial properties through its lysine residues (Beck et al., 2013). Another upregulated protein, the pentraxin serum amyloid P, was found to regulate the immune system by activating the complement pathway, decreasing neutrophil adhesion, regulating macrophage activation, and enhancing phagocytosis (Cox et al., 2014). Upregulation of haptoglobin and hemopexin in poly(I:C) MSC-EVs may enhance antimicrobial activity by accelerating the clearance of free hemoglobin and heme, thereby limiting the availability of iron necessary for bacterial growth (Zauberman et al., 2019). Interestingly, alpha-2-macroglobulin, an endogenous protein present in human saliva, nasal secretions, and blood, and also found to be elevated in poly(I:C) MSC-EVs, was shown to possess broad-spectrum anti-influenza activity (Chen et al., 2010).

Poly(I:C) priming also increased the abundance of EV proteins that have demonstrated antibiofilm activity. Infections associated with drug-resistant biofilms are a global health concern, having a tremendous impact on mortality and quality of life and creating an economic burden for healthcare systems worldwide (Wu et al., 2019). Serum paraoxonase/arylesterase 1 (PON1) is a lactonase that has been proposed as a potential antibiofilm agent because it can hydrolyze acyl homoserine lactones, which are major quorum-sensing signals in Gram-negative bacteria important during biofilm formation (Camps et al., 2011). Serum amyloid A is an acute phase protein protective against bacterial, fungal, and viral pathogens that was shown to prevent biofilm formation *in vitro* by uropathogenic *E. coli* (Erman et al., 2012). Some of the identified EV proteins whose levels were not altered by poly(I:C) preconditioning also have been shown to possess antibiofilm activity including lactoferrin, neutrophil defensin 1, and S100A8/A9 (Trøstrup et al., 2017; Moazzezy et al., 2020; Ramamourthy and Vogel, 2020).

Findings from this study also showed that poly(I:C) priming appears to have a greater effect on EV protein cargo than EV miRNA cargo, suggesting that the enhanced antimicrobial effects observed after poly(I:C) treatment is not due to altered EV miRNA content (Monsel et al., 2015; Park et al., 2019). The effect of priming was also found to be considerably less important on EV miRNA than protein content in other studies that investigated the effects of IFNg (Marinaro et al., 2019; Peltzer et al., 2020).

Although we were unable to demonstrate that poly(I:C) preconditioning significantly altered MSC-EV miRNA content, it is likely that EV-mediated transfer of miRNAs released from MSCs in the presence or absence of poly(I:C) stimulation may influence innate immunity *via* regulation of proinflammatory and anti-inflammatory cytokine production and antimicrobial responses (Bulut and GÜrsel, 2020). Several of the identified abundant EV miRNAs have been shown to act as regulators of immune and inflammatory responses including miR-21-5p, let-7f, miR-199a, miR-221, miR-423-5p, miR-409-3p, miR-181a-5p, miR-22, miR-100-5p, miR-30a-5p, and miR-31-5p (Sheedy, 2015; Qian et al., 2016; Wan et al., 2016; Zhu et al., 2017; Liu et al., 2019; Luo et al., 2019; Wang et al., 2019; Jiang et al., 2020; Li et al., 2021). Analysis of predicted gene targets of these highly expressed miRNAs identified several signaling pathways important in innate immunity including the MAPK signaling pathway, PI3K-Akt signaling pathway, chemokine signaling pathway, transforming growth factor beta signaling pathway, and the TLR signaling pathway. The PI3-Akt signaling pathway has previously been reported to be among the top 10 signaling pathways in MSC-EVs and is known to play critical regulatory roles in various MSC functions including survival, migration, proliferation, cytokine production, differentiation, and angiogenesis (Mardpour et al., 2019).

We detected miR-21-5p as the most highly expressed miRNA in MSC-EVs regardless of poly(I:C) preconditioning. MiR-21 has been shown to play a dynamic role in inflammation, controlling the balance between proinflammatory and immunosuppressive responses and has demonstrated antibacterial, anti-inflammatory, and proliferative roles in a diabetic infected wound model (Sheedy, 2015; Li et al., 2019). The delivery of miR-21-5p by secreted EVs has been suggested to be a potential mechanism responsible for the immunomodulatory effects of MSC-EVs observed on dendritic cell maturation and function, causing reduced production of proinflammatory cytokines IL-6 and IL-12 and decreased CCR7 expression important for dendritic cell homing to lymph nodes (Reis et al., 2018). Dendritic cells are known to play a critical role in initiating and regulating immune responses and they are a key target for MSC-mediated immunomodulation (Reis et al., 2018). Although we did not observe increased miR-21-5p expression in poly(I:C) MSC-EVs, Fukushima et al. (2018) found that poly(I:C) stimulation increased miR-21 levels in EVs released from various human cell lines. However, that study exposed cells to 50 μg/ml poly(I:C) for 24 h, while our study utilized a concentration of 1 μg/ml poly(I:C) for 1 h. Waterman et al. (2010) previously determined that the low dose of 1 μg/ml poly(I:C) for 1 h was essential to achieve the immunosuppressive phenotype of the MSCs, which mimics the gradient of danger signals endogenous MSCs encounter and respond to at a distance from the site of injury. Our laboratory verified TLR3 activation in poly(I:C)-treated MSCs by demonstrating an upregulation of CXCL10 (6.5-fold increase, *p* = 0.07) and IL-8 (3.7-fold increase, *p* = 0.003) mRNA expression in MSCs after 1 h of treatment (not shown). Tomchuck et al. (2008) showed that CXCL10 and IL-8 were among the most highly expressed TLR3-regulated genes in human MSCs induced by poly(I:C) stimulation.

Limitations of this study exist. Future experiments are needed to validate differentially expressed proteins and to analyze functional protein content of MSC-EVs *in vitro*. The antimicrobial properties of the MSC-EVs with and without poly(I:C) priming should also be verified *in vivo* using animal models. In addition, phenotyping of EVs was performed in this investigation by nano-flow cytometry using antibodies against surface proteins CD9 and CD81 and not by Western blot analysis, although it has been suggested that nano-flow cytometry provides quantitative analysis of EV size distribution, concentration, purity, as well as phenotype without bias (Tian et al., 2019).

## Conclusion

As antibiotic resistance continues to increase globally, there is an urgent need for novel, non-antibiotic approaches to control chronic drug-resistant infections. In this study, we determined that preconditioning bone marrow-derived MSCs with the TLR3 agonist poly(I:C) enhances the antimicrobial and immunomodulatory proteomic profile of secreted EVs without significantly altering EV miRNA content. These results suggest that poly(I:C) MSC-EVs have immense potential as an allogeneic, “off-the-shelf” cell-free therapy for infectious diseases, offering the benefits of stem cell therapy while representing a theoretically safer alternative. Further investigation is vastly needed using MSCs from additional donors to determine the relative importance of particular functional EV proteins and their activated signaling pathways following MSC-EV interaction with immune cells.

## Data Availability Statement

The datasets presented in this study can be found in online repositories. The names of the repository/repositories and accession number(s) can be found below: ProteomeXchange Consortium *via* the PRIDE partner repository with the dataset identifier PXD024790 and doi: 10.6019/PXD024790, RNA-seq data has been deposited in the Sequence Read Archive (SRA) at https://www.ncbi.nlm.nih.gov.sra/PRJNA708250 accession numbers SRX10291982, SRX10291983, SRX10291984, SRX10291985, SRX10291986, and SRX10291987.

## Author Contributions

LP and WK conceived and designed the experiments and reviewed and edited the manuscript. WK performed the experiments. LP analyzed the data and wrote the manuscript. Both authors contributed to the article and approved the submitted version.

## Conflict of Interest

The authors declare that the research was conducted in the absence of any commercial or financial relationships that could be construed as a potential conflict of interest.
