# Supplementary material for: Priming With Toll-Like Receptor 3 Agonist Poly(I:C) Enhances Content of Innate Immune Defense Proteins but Not MicroRNAs in Human Mesenchymal Stem Cell-Derived Extracellular Vesicles
Source: Front Cell Dev Biol. 2021 May 24;9:676356. doi: 10.3389/fcell.2021.676356 (PMC8180863; doi:10.3389/fcell.2021.676356)
Supplement: Supplementary file 3 [file Data_Sheet_1.PDF]

Supplementary Material

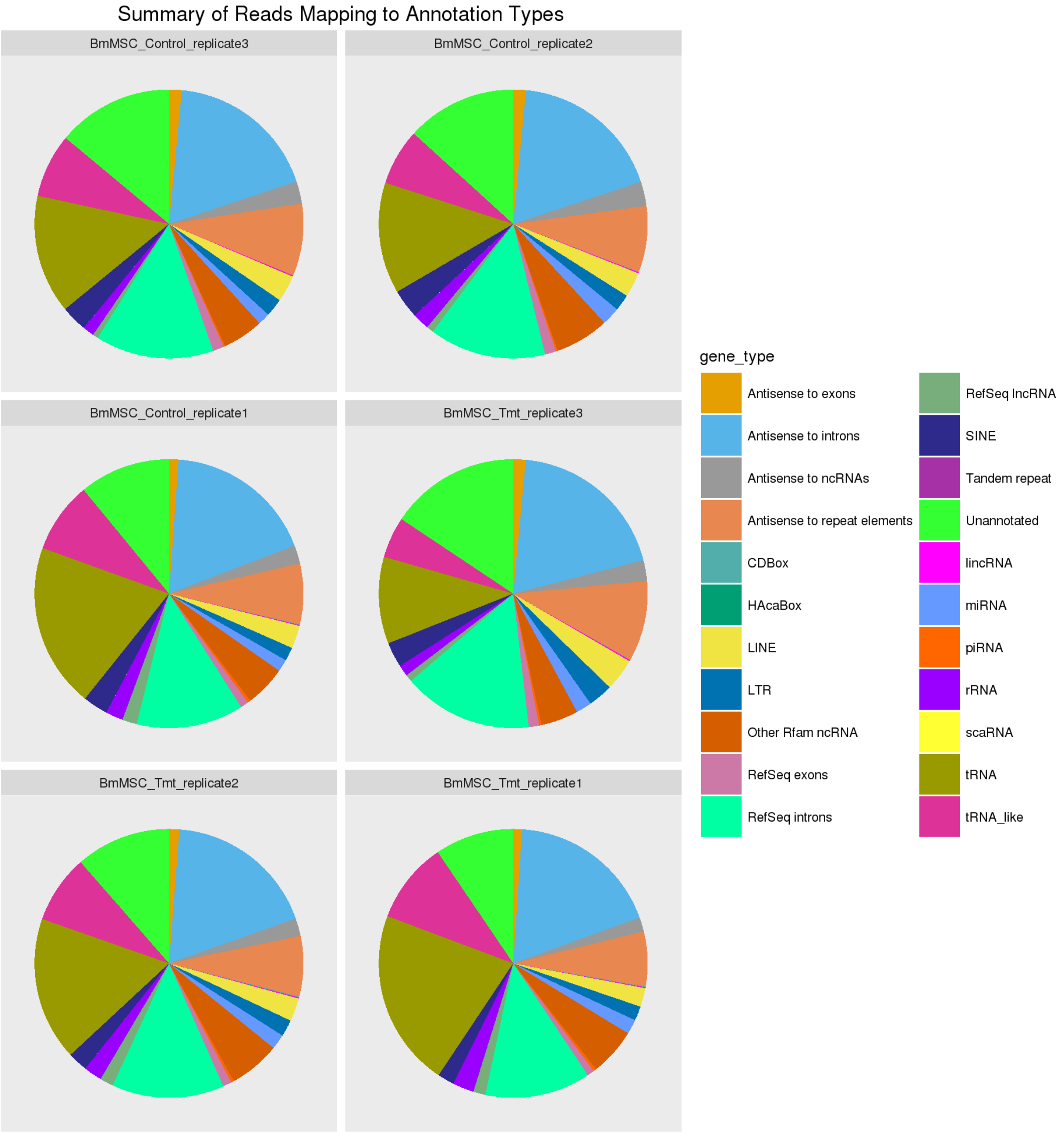

Supplementary Figure 1. Small RNA expression summary in control and poly(I:C)-primed MSC-EVs. Pie charts summarize the number of reads mapping to each annotation type in each replicate. BmMSC, bone marrow-derived MSCs; Tmt, treatment with poly(I:C).

Supplementary Tables

Supplementary Tables 1 and 3 have been uploaded and are available as Data Sheets (excel files).

Supplementary Table 1. List of 238 proteins that were identified in all EV replicates ± poly(I:C).

Supplementary Table 2. Functional Annotation Clustering analysis of proteins identified in all EV replicates ± poly(I:C).

Supplementary Table 3. List of 250 miRNAs that were identified in all EV replicates ± poly(I:C).

Supplementary Table 4. The 25 most abundant miRNAs identified in EVs ± poly(I:C). Those miRNAs which were also found to be among the most abundant in human bone marrow-derived MSC-EVs by Baglio et al. (2015) and Ferguson et al. (2018) are highlighted in bold.

Supplementary Table 5. KEGG signaling pathways enriched among the genes predicted to be targeted by the 25 most abundant miRNAs identified in EVs ± poly(I:C).

Supplementary Table 2. Functional Annotation Clustering analysis of proteins identified in all EV replicates ± poly(I:C).

Term	Count	%	P Value	Benjamini P value
Functional Annotation Cluster 1 Enrichment Score: 17.49				
GO:0005615 extracellular space	99	50.3	2.44E-58	3.31E-56
UP_KEYWORDS secreted	74	37.6	6.08E-26	1.74E-23
UP_SEQ_FEATURE signal peptide	77	39.1	3.79E-14	4.57E-11
UP_KEYWORDS glycoprotein	77	39.1	9.04E-08	1.18E-06
Functional Annotation Cluster 2 Enrichment Score: 11.19				
GO:0010951 negative regulation of endopeptidase activity	21	10.7	5.93E-18	4.02E-15
UP_KEYWORDS serine protease inhibitor	15	7.6	7.87E-14	3.92E-12
Functional Annotation Cluster 3 Enrichment Score: 7.09				
GO:0005839 proteasome core complex	10	5.1	4.21E-13	1.43E-11
GO:0038061 NIK/NF-kappaB signaling	11	5.6	3.48E-09	5.90E-07
GO:0002479 antigen processing and presentation of exogenous peptide antigen via MHC class I, TAP-dependent	10	5.1	3.68E-08	3.12E-06
GO:0060071 Wnt signaling pathway, planar cell polarity pathway	11	5.6	9.20E-08	6.94E-06
GO:0033209 tumor necrosis factor-mediated signaling pathway	11	5.6	9.64E-07	5.45E-05
GO:0000165 MAPK cascade	12	6.1	2.10E-04	0.0059
UP_KEYWORDS host-virus interaction	13	6.6	3.37E-04	0.0024
Functional Annotation Cluster 4 Enrichment Score: 6.04				
GO:0098641 cadherin binding involved in cell-cell adhesion	19	9.6	4.60E-09	6.40E-07
GO:0098609 cell-cell adhesion	14	7.1	1.40E-05	6.12E-04
Functional Annotation Cluster 5 Enrichment Score: 3.90				
GO:0030216 keratinocyte differentiation	8	4.1	2.49E-05	9.64E-04
GO:0001533 cornified envelope	6	3.0	1.31E-04	0.0017
Functional Annotation Cluster 6 Enrichment Score: 3.81				
UP_KEYWORDS innate immunity	12	6.1	4.33E-05	4.13E-04
UP_KEYWORDS immunity	16	8.1	9.41E-05	7.91E-04
Functional Annotation Cluster 7 Enrichment Score: 3.50				
GO:0098869 cellular oxidant detoxification	8	4.1	1.44E-05	6.12E-04
Functional Annotation Cluster 8 Enrichment Score: 3.23				
GO:0042730 fibrinolysis	9	4.6	4.34E-11	9.81E-09
KEGG_PATHWAY hsa04610:complement and coagulation cascades	14	7.1	1.22E-10	1.93E-08
UP_KEYWORDS hemostasis	9	4.6	1.60E-08	2.39E-07
Functional Annotation Cluster 9 Enrichment Score: 2.72				
UP_KEYWORDS antimicrobial	8	4.1	7.90E-05	7.07E-04
GO:0050832 defense response to fungus	4	2.0	0.0035	0.065
GO:0042742 defense response to bacterium	6	3.0	0.025	0.29
Functional Annotation Cluster 10 Enrichment Score: 2.63				
GO:0006957 complement activation, alternative pathway	4	2.0	3.81E-04	0.0098
GO:0006958 complement activation, classical pathway	7	3.6	9.34E-04	0.021

Supplementary Table 4. The 25 most abundant miRNAs identified in MSC-EVs ± poly(I:C). Those miRNAs which were also found to be among the most abundant in human bone marrow-derived MSC-EVs by Baglio et al. (2015) and Ferguson et al. (2018) are highlighted in bold.

miRNA	Chromosome
**hsa-miR-21-5p**	chr17
**hsa-miR-221-3p**	chrX
**hsa-let-7b-5p**	chr22
**hsa-let-7a-5p**	chr11
hsa-miR-320a-3p	chr8
**hsa-miR-143-3p**	chr5
**hsa-miR-199a-3p**	chr1
**hsa-miR-199b-3p**	chr9
**hsa-let-7i-5p**	chr12
hsa-miR-24-3p	chr19
hsa-let-7e-5p	chr19
**hsa-miR-222-3p**	chrX
**hsa-let-7f-5p**	chr9
hsa-miR-181a-5p	chr1
**hsa-miR-22-3p**	chr17
hsa-miR-30a-5p	chr6
**hsa-miR-423-5p**	chr17
hsa-miR-320b	chr1
hsa-miR-409-3p	chr14
hsa-miR-432-5p	chr14
**hsa-miR-27b-3p**	chr9
hsa-miR-493-5p	chr14
**hsa-miR-100-5p**	chr11
hsa-miR-31-5p	chr9
**hsa-miR-92a-3p**	chr13

Supplementary Table 5. KEGG signaling pathways enriched among the genes predicted to be targeted by the 25 most abundant miRNAs identified in EVs ± poly(I:C).

Term	Count	Fold Enrichment	P Value	Benjamini P value
KEGG_PATHWAY hsa04010:MAPK signaling pathway	54	2.27	1.19E-08	1.63E-06
KEGG_PATHWAY hsa04015:Rap1 signaling pathway	43	2.17	1.52E-06	1.36E-04
KEGG_PATHWAY hsa04151:PI3K-Akt signaling pathway	58	1.78	1.30E-05	8.74E-04
KEGG_PATHWAY hsa04014:Ras signaling pathway	41	1.93	5.59E-05	0.0025
KEGG_PATHWAY hsa04062:chemokine signaling pathway	35	2.00	1.05E-04	0.0035
KEGG_PATHWAY hsa04350:TGF-beta signaling pathway	20	2.53	2.25E-04	0.0062
KEGG_PATHWAY hsa04012:ErbB signaling pathway	20	2.44	3.64E-04	0.0089
KEGG_PATHWAY hsa04068:FoxO signaling pathway	25	1.98	0.0014	0.021
KEGG_PATHWAY hsa04722:neurotrophin signaling pathway	23	2.03	0.0016	0.023
KEGG_PATHWAY hsa04620:toll-like receptor signaling pathway	21	2.10	0.0018	0.024
KEGG_PATHWAY hsa04915:estrogen signaling pathway	20	2.14	0.0019	0.024
KEGG_PATHWAY hsa04152:AMPK signaling pathway	23	1.99	0.0023	0.027
KEGG_PATHWAY hsa04910:insulin signaling pathway	24	1.85	0.0046	0.042
KEGG_PATHWAY hsa04390:hippo signaling pathway	25	1.76	0.0070	0.054
KEGG_PATHWAY hsa04071:sphingolipid signaling pathway	21	1.86	0.0079	0.056
KEGG_PATHWAY hsa04310:Wnt signaling pathway	23	1.77	0.0093	0.064
KEGG_PATHWAY hsa04668:TNF signaling pathway	19	1.89	0.010	0.068
KEGG_PATHWAY hsa04919:thyroid hormone signaling pathway	20	1.85	0.010	0.068
KEGG_PATHWAY hsa04917:prolactin signaling pathway	14	2.09	0.014	0.079
KEGG_PATHWAY hsa04921:oxytocin signaling pathway	23	1.63	0.023	0.11
KEGG_PATHWAY hsa04660:T cell receptor signaling pathway	17	1.80	0.024	0.11
KEGG_PATHWAY hsa04370:VEGF signaling pathway	12	2.09	0.025	0.12
KEGG_PATHWAY hsa04550:signaling pathways regulating pluripotency of stem cells	21	1.59	0.038	0.15
KEGG_PATHWAY hsa04150:mTOR signaling pathway	11	2.01	0.042	0.16
KEGG_PATHWAY hsa04024:cAMP signaling pathway	27	1.45	0.049	0.19
